# Crayfish Eating in Snakes: Testing How Anatomy and Behavior Affect Prey Size and Feeding Performance

**DOI:** 10.1093/iob/obab001

**Published:** 2021-01-30

**Authors:** N D Gripshover, B C Jayne

**Affiliations:** 1 Department of Biological Sciences, University of Cincinnati, PO Box 210006, Cincinnati, OH 45221-0006, USA; 2 Department of Biological Sciences, Florida International University, Miami, FL 33199, USA

## Abstract

Quantifying the performance of animals is a powerful methodology for determining the functional consequences of morphological variation. For example, snakes consume prey whole, and variation in the anatomy of their trophic apparatus directly affects gape and limits maximal prey size. However, for the foraging ecology of snakes and other systems, scant data exist regarding how often maximal capacities are taxed in nature. Hence, we quantified: (1) maximal gape, (2) the size of prey relative to maximal gape, and (3) how the type and relative size of prey affected behavior and prey handling times (HTs) for two species of natricine snakes that primarily eat soft- (*Regina septemvittata*) or hard-shelled (*Liodytes alleni*) crayfish. *Liodytes alleni* had significantly larger maximal gape than *R. septemvittata* with equal snout–vent length. The percentages of large prey (>60% maximal gape area) consumed in the field were low in both *R. septemvittata* (22%) and *L. alleni* (2%). However, *R. septemvittata*, especially juveniles, ate relatively larger prey than *L. alleni*. Strategies for dealing with the seasonal scarcity of small crayfish differed as juvenile *R. septemvittata* commonly removed and ate only chelipeds from crayfish too large to swallow whole, whereas juvenile *L*. *alleni* ate many small odonate nymphs. During laboratory trials, unlike *R. septemvittata*, *L. alleni* usually used its body to restrain prey with behaviors that depended on relative prey size and prey hardness. *Liodytes alleni* consumed soft-shelled crayfish significantly faster than *R. septemvittata* and significantly faster than hard-shelled crayfish. Several of the differences in gape, prey size, and prey HTs and behavior between the crayfish-eating snakes resemble those between two phylogenetically distant species of homalopsid snakes that consume either hard- or soft-shelled crabs. In both groups of crustacean-eating snakes, the decreased capture success in captivity and the rare consumption of relatively large hard-shelled crustaceans in the field suggest that the ability to capture this type of prey constrains prey size more commonly than maximal gape. Based on data integrating snake size and gape with the relative mass of intact prey, the predicted potential feeding performance *R. septemvittata* consuming intact prey exceeded that of the other three species.

## Introduction

Quantifying the performance of animals is widely recognized as a powerful methodology for determining the functional consequences of morphological variation. Although laboratory tests have been useful and common, quantifying the performance of animals in nature is usually difficult and hence much less common (Irschick and Higham 2016). Consequently, the question of whether or not animals are “olympians” that routinely tax their maximal capacities in nature (Hertz et al. 1988) has rarely been addressed with empirical data either for individuals within a species or between different species. As a result of their environment and their behavior, the performance of some animals in nature may be considerably less than their maximal capabilities (Irschick and Higham 2016) just as realized niches are often small compared with the fundamental niches of animals (McGill et al. 2006). However, some species have higher performance in nature than under presumably optimal laboratory conditions (Jayne and Ellis 1998). This primacy of behavior for affecting ecological performance is emphasized further by some systems in which individuals with inferior laboratory performance outperform other conspecific individuals with higher laboratory performance as a result of using a greater fraction of their maximal capacities when in the field (Husak and Fox 2006).

The feeding of snakes has several attributes well suited for employing the performance testing paradigm to gain fundamental insights into the roles of morphology and behavior for affecting foraging ecology. For example, even though determining the maximal gape of snakes requires accounting for the mobility and dimensions of the relevant bones and the distensibility of soft tissues (Cundall 2019), this is still much less complex than traits such as maximal locomotor speed which have effects of morphology that are mediated by muscle physiology, neural control, the motivation of the test subject, and environmental conditions (Irschick and Higham 2016). Consequently, the gape of snakes was a prominent example in a seminal paper that developed the morphology–performance–fitness paradigm (Arnold 1983). Ironically, maximal gape has only recently been measured directly in less than 10 of more than 3,500 species of snakes with diverse ecology and morphology (Hampton and Moon 2013; Hampton and Kalmus 2014; Hampton 2018; Jayne et al. 2018), and accompanying data on relative prey size are only available for three species (Jayne et al. 2018). Consequently, relating the size of prey to the maximal gape to the feeding behavior and ecology of snakes remains in its infancy.

Additional advantages of studying foraging ecology using snakes are that determining the size and identity of prey is facilitated because snakes consume their prey whole (Voris and Voris 1983; Mushinsky 1987), and large samples of stomach contents often can be obtained with non-destructive sampling (Mushinsky et al. 1982; Glaudas et al. 2019). Consequently, many studies of snakes have documented the types and sizes of prey and how they are related to the lengths and masses of snakes (Glaudas et al. 2019). Overall, larger snakes eat larger prey, and larger individuals commonly do not consume prey as small as those of smaller conspecifics (Mushinsky 1987; Arnold 1993). However, such previous analyses of the sizes of predators and prey do not empirically address the role of anatomical constraints in affecting prey size.

Additional metrics of feeding performance besides prey size include the ease of capture and prey handling time (HT), which are affected by the type of prey (Arnold 1993; Willson and Hopkins 2011). For example, many snakes with the generalized morphology of slender, pointed teeth adeptly eat prey such as earthworms, amphibians, fishes, and mammals, whereas snakes that eat hard-bodied prey often have reduced tooth length and sharpness (Savitzky 1983), suggesting that such prey are difficult for more generalized snakes to exploit. Their hard and spiny exoskeletons and powerful chelae make many crustaceans formidable prey for snakes (Godley 1980). Nonetheless, current phylogenies (Figueroa et al. 2016) suggest that crustacean-eating specialists have evolved independently in the New World natricine and southeast Asian homalopsid snakes, with some genera specializing in consuming either freshly molted (*Gerarda*, *Regina*) or hard-shelled crustaceans (*Fordonia*, *Liodytes*) (Franz 1977; Gibbons and Dorcas 2004; Jayne et al. 2018). This presents an unusual opportunity to examine the extent to which convergent ecology is correlated with convergent evolution of morphology, feeding behavior, and performance.

We studied two species of crayfish-eating snakes, *Regina septemvittata* and *Liodytes alleni* to test how an anatomical constraint (maximum gape) and prey choice interact with prey handling behaviors to affect diet and feeding performance within and between species. We quantified the scaling relationships between maximum gape and overall snake size, and for snakes captured in the field we determined the size of prey relative to maximal gape. Determining gape allowed us to test the extent to which snakes in the field use their maximal capacity for swallowing prey and how relative prey size affects behavior and prey HTs (observed in laboratory experiments). The reduced hardness and mobility of soft-shelled crayfish and previous findings for crustacean-eating homalopsid snakes (Jayne et al. 2018) led us to hypothesize that *R. septemvittata* would consume soft-shelled crayfish more rapidly and select prey with larger relative size in the field than *L. alleni*. Finally, to gain insights into convergence or the lack thereof between different lineages of snakes that independently became crustacean-eating specialists, we compared their behaviors and feeding performance using new methods that integrate the scaling relationships of both prey and predator size with the constraint of gape on prey size.

## Materials and methods

### Study species and field observations

All snakes in our study were from wild populations. Between April and October in 2018 and 2019, we collected *R. septemvittata* and co-occurring crayfish, *Orconectes rusticus*, by overturning rocks along a stream in Kenton Co., Kentucky (KDFWR permit SC1711317 and SC1911197). During May 2019 in Gilchrist and Levy Counties in Florida, we collected *L. alleni* and co-occurring crayfish, *Procambarus fallax*, by seining mats of aquatic plants (*Limnobium spongia*) using the same methodology as Godley (1980) (FWC permit LSSC-18-00055A). We retained some gravid *L. alleni* to obtain neonatal snakes for measuring maximal gape. All of the husbandry and experimental methods were in compliance with the IACUC of the University of Cincinnati.

To collect stomach contents from snakes captured in the field, we were able to gently palpate the stomachs of *R. septemvittata* to force regurgitation. After measuring snout–vent length (SVL) and mass, we released these snakes at their site of capture. Unlike *R*. *septemvittata*, *L. alleni* cannot be forced to regurgitate their hard and spiny prey without severely injuring or killing the snake (Godley 1980). Consequently, we only used palpation to detect stomach contents in the *L. alleni* (*n *=* *17) that we captured, and we subsequently euthanized these snakes to recover their stomach contents. Furthermore, unlike *R*. *septemvittata*, *L*. *alleni* has some variation in diet (consumption of odonates and shrimp) that occurs seasonally and ontogenetically. Thus, to increase the sample size of prey for *L*. *alleni* without killing more animals and to obtain a sample over a greater span of the active season, we analyzed stomach contents from 27 snakes that were collected by Godley (1980) in Glades County Florida and are now in the collection of the National Museum of Natural History (Suitland, MD, USA).

### Gape and prey size

We measured gape for the following samples that ranged from neonates to large adults. The 15 male and 12 female *R. septemvittata* had overall means±SE of SVL and mass of 317 ± 24.5 mm (range = 155–586 mm) and 21.9 ± 4.6 g (range = 1.7–97.6 g), respectively. The 6 male and 24 female *L. alleni* had overall mean values of SVL and mass of 243 ± 20.3 mm (range *=* 120–490 mm) and 26.7 ± 6.3 g (range = 2.0–129 g), respectively. Besides including neonatal snakes for both study species, the large individuals in our sample were a sizable fraction of values reported for maximal sizes in different collections of *R*. *septemvittata* (SVL = 540–720 mm) and *L*. *alleni* (SVL = 540 mm) (Gibbons and Dorcas 2004). For all of these preserved specimens, we used the non-gravid masses of females.

Before measuring gape, we euthanized the snakes by injecting them with an overdose of sodium pentobarbital. We then made a transverse incision through all of the skin and structures ventral to the vertebrae approximately two skull lengths posterior to the skull. This transverse incision prevented the probe from getting snagged by the tips of the recurved teeth as it allowed a one-way (posterior) movement of the probe as it was inserted into the mouth and then removed through the incision in preparation for the next largest probe. The probes for measuring gape, which were 3D-printed, were cylinders with a hemispherical end with a diameter equal to that of the cylinder. Before inserting the hemispherical end into the mouth of the snake, the probe was lubricated with KY Jelly to simulate the lubricating effect of saliva.

The first probe inserted into the snake had a diameter less than the width of the snake’s head, and its insertion encountered very little resistance. We then proceeded to insert successively larger probes. The successive incremental increases in probe diameter were 0.5 and 1.0 mm for probes with diameters less than or greater than 11 mm, respectively. The minimum and maximum gape diameter measured was 8 and 24 mm, respectively. For diameters from 8 to 10.5 mm, the incremental increases in diameter and area ranged from 6.3% to 4.8%, and 12.9% to 9.8%, respectively. For diameters from 11 to 23 mm, the incremental increases in diameter and area ranged from 9.1% to 4.3%, and 19.0% to 8.9%, respectively.

**Fig. 1:**
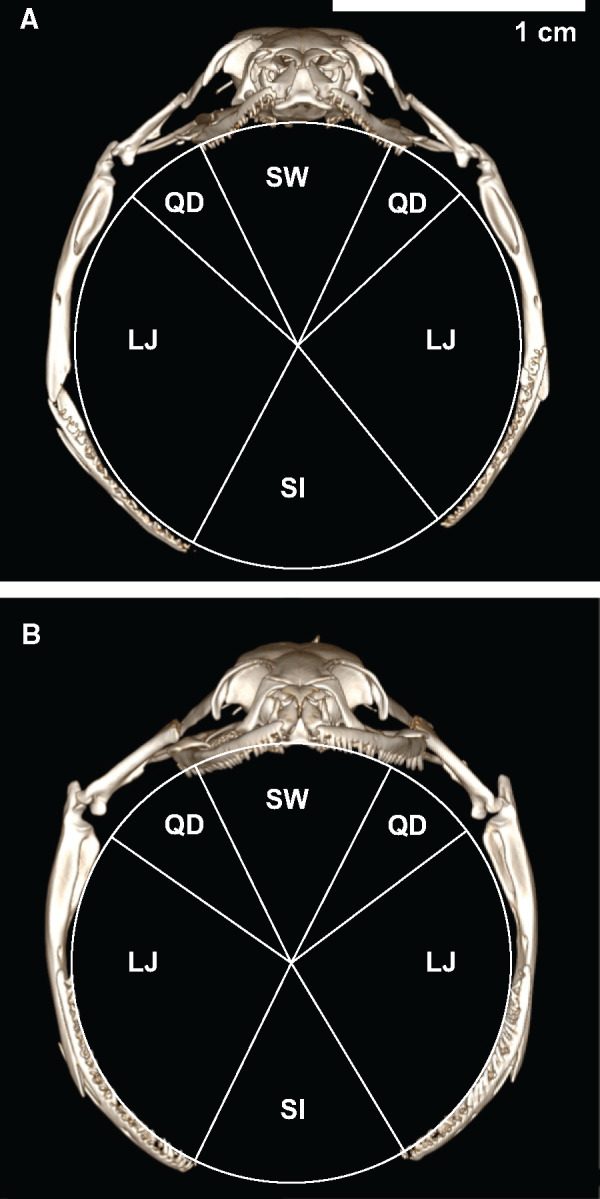
Contributions of skeletal elements and soft tissues to maximal gape area. Anterior views of computed tomography scans of *R. septemvittata* (**A**) and *L. alleni* (**B**) preserved at maximum gape. The relative contributions to maximal gape area are shown for the skull width (SW), quadrate (QD), lower jaw (LJ), and the skin and other intermandibular soft tissues between the lower jaws (SI). See Table 2 for mean values and comparisons between species.

Snakes require a considerable amount of time to eat very large prey as some of the HTs of our captive snakes exceeded 30 min. Thus, to better simulate these conditions when resistance to insertion increased markedly (usually for the last three or four probes), we waited 10 min before inserting the next probe. We inserted larger probes until they did not fit or until we observed tissue failure (such as torn skin), after which we reinserted the next smaller probe (= maximal gape). Based on external appearance, the limiting factor for gape was usually the skin behind the head rather than the structures forming the margins of the mouth opening. At maximal gape the skin of the neck was often so tight that it could not be moved noticeably relative to the underlying probe, whereas such movement was often possible for the skin that was between the lower jaws. Only in *R*. *septemvittata*, CT scans of some specimens revealed that one of the dentary bones seemed displaced to an unusual extent relative to the more proximal bones (Fig. 1A), and the arc lengths of these displacement approximated 3–4% of the circumference of the probe. Given these values and the incremental increases in probe size, we suspect that such displacement arose from inserting the largest probe and then remained in place after the next to largest probe was reinserted. Hence, a variety of measurement errors could easily affect measurement of gape area by 5–10%.

A 3D-printed plastic cylinder with a diameter equal to the maximal gape remained in the mouth when the specimen was fixed with 10% formalin and subsequently stored in 70% ethanol. Of these preserved specimens, we used 10 *R*. *septemvittata* (SVL = 163–586 mm) and 9 *L*. *alleni* (SVL = 140–473 mm) to obtain CT scans that were subsequently used to determine the contributions of different structures to maximal gape area. From anterior view images of these CT scans, we located: (1) the joints between the supratemporal bone and the quadrate, (2) the joints between the quadrate and the lower jaw, and (3) the tips of the lower jaws. We then used a graphical program to construct lines that passed though these anatomical landmarks and the center of the circle created by the plastic cylinder in the mouth of the snake. For the resulting angles defined by these reference lines, we determined the fraction of the circular area that were accounted for by the: (1) width of the skull (measured between the supratemporal-quadrate joints, (2) the lengths of the quadrates, (3) lengths of the lower jaws (from the tips to the center of the joint with the quadrate), and (4) length of the skin and other soft tissues between the lower jaws (Fig. 1).

We converted the maximal cross-sectional areas and intact masses of all prey to values of relative prey area (RPA) and relative prey mass (RPM) that were percentages of the maximal gape area and snake mass, respectively. For nearly all of the *R*. *septemvittata* captured and released in the field and for all the museum specimens of *L*. *alleni*, the values of maximal gape area used for RPA were predicted from the relevant scaling equations with the SVL of the snakes. To estimate the intact size of stomach contents, we used regressions from reference specimens of 66 *O. rusticus* (mass 0.13–12.88 g) and 44 *P. fallax* (mass 0.19–14.32 g) that related carapace and chela dimensions to maximal cross-sectional area calculated from an ellipse where maximal height and width of the carapace formed the axes. The crayfish from stomachs were only classified as either soft- or hard-shelled rather than making finer distinctions, such as pre-molt as in Godley (1980).

### Laboratory experiments

We housed and tested snakes individually in 10-gallon glass aquariums with 25-W incandescent light bulbs that allowed the snakes to thermoregulate body temperatures from approximately 25–30°C, which is within the range of active field body temperatures. For example, 233 *R*. *septemvittata* that we captured had a mean body temperature of 27.6°C (SE = 0.2; range = 15.1°–34.5°), and Godley (1980) found prey in *R*. *alleni* captured where water temperature ranged from 15°C to 27°C. The aquariums were tilted so that a pool of deionized water in the lower half of the container had a maximum depth of 3 cm. The lower half of the tank was covered with a layer of smooth pebbles (Imagitarium River Rock Shallow Creek Gravel, Petco, San Diego, CA, USA), whereas the upper half was covered with artificial turf.

For captive feedings the crayfish often molted at unknown times overnight as much as 12 h before the feeding. To store calcium prior to molting, crayfish grow a gastrolith (McWhinnie 1962), which is subsequently resorbed as the crayfish harden. To gain further insights into the rate of the hardening process after our feeding experiments were completed, we developed a system in which *O. rusticus* were maintained with identical conditions as those in our feeding experiments, and then we preserved these crayfish over a wide range of known post-molt times (0.1–36 h). For *O*. *rusticus* with masses ranging from 0.5 to 2 g, the earliest times at which the gastrolith was too small to be found in a dissection with a dissecting microscope were between 26 and 30 h. Thus, post-molt hardening is a prolonged and continuous process largely lacking discrete changes that are evident from handling an intact crayfish. However, for a short time after molting (usually < 4 h) the abilities to right and move out of water are reduced, even though crayfish can walk readily in water and perform a tail flip escape response. Crayfish with these characteristics were categorized as “soft” (numerical score of 0). All other recently molted crayfish that had greater mobility and hardness as much a 15 h post molt were categorized as having “medium” hardness (numerical score of 1), and we used “hard” to refer to crayfish in the laboratory that were at least 4 days post molt (numerical score of 3).

From HD (1920 × 1080 pixels) videos of captive feedings, we determined total HT as the sum of the durations of the following six behaviors. (1) Attack extended from the first strike until the final strike that succeeded in capturing the crayfish. (2) Jaw holding occurred immediately after the successful strike as snakes grasped prey with immobile jaws. (3) Lateral jaw walking movements repositioned the prey (without engulfing it) prior to the beginning of swallowing. These movements usually involved moving the head of the snake along the long axis of the prey so that swallowing would start at either the posterior or anterior end of the crayfish. (4) Pauses occurred during lateral jaw walking. (5) Swallowing ended as soon as jaw movements ceased and the prey item was no longer visible. (6) Pauses occurred during swallowing.

We noted the following additional events. We scored the number of successful and unsuccessful strikes, the number of escapes by the crayfish, and the snake behaviors during which the escapes occurred. We scored the following four locations of the final strike that succeeded in capturing and consuming the crayfish: (1) completely posterior to the carapace, (2) the joint between the abdomen and carapace, (3) the carapace only, and (4) the cheliped. Immediately after a successful strike, we recorded whether the back of the mouth of the snake (the joints between the quadrate and lower jaw) was located on the lateral, dorsal, or ventral surface of the crayfish. To facilitate statistical analyses we encoded numerical values of 0 and 1 for swallowing prey tail first or head first, respectively, and values of 1, 2, and 3 for the dorsal, lateral, or ventral surface of the prey begin oriented toward the palate, respectively. For *L*. *alleni*, we recorded the type and total duration of body restraint used by the snakes. We also recorded the occurrence of tail flip escape responses by the crayfish and the number of times the crayfish pinched the snake to determine whether or not these behaviors were associated with escapes or had significant effects on HT.

### Statistical analysis

To quantify scaling relationships, all morphological data and HTs were log10 transformed before they were analyzed. In both of our study species, females attain larger overall size than males (Gibbons and Dorcas 2004). Unless stated otherwise, we found no significant differences between males and females within each species, and the least-squares scaling regressions and other analyses were performed for a combined sample of males and females. We used analyses of covariance (ANCOVA) to compare regressions between species, and unless stated otherwise, these comparisons lacked significant heterogeneity of slopes. To determine the extent to which a maximal capacity (gape) was used by animals in nature, we generated frequency distributions of RPA for items recovered from the stomachs of snakes captured in the field. To compare these cumulative frequency distributions of RPA between species, we used a two-sample Kolmogorov–Smirnov Test. To test how prey HTs during laboratory trials were affected by variables such as RPA, strike location, swallowing direction, and prey orientation, we used forced-fit multiple regressions with these quantities as independent variables. Our final choice of a model was one for which all independent variables were individually significant and the highest *R*^2^ value was attained. We also compared regressions of HT versus RPA to make comparisons among different species of crustacean-eating specialists as well as between hard- and soft-shelled prey. Means are reported ±SE. Tabular summaries of regression analyses, and ANCOVAs are provided in the Supplementary Materials (Supplementary Tables S1–S5).

## Results

### Morphology

**Fig. 2:**
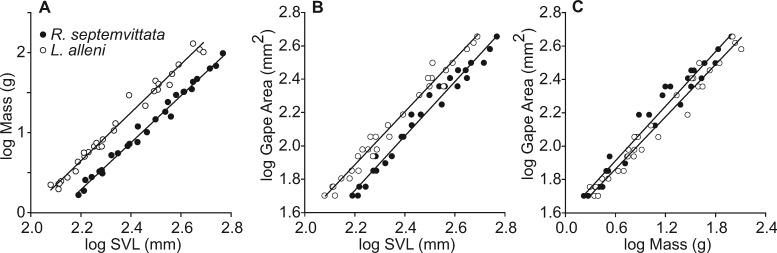
Scaling relationships for morphological data of *R. septemvittata* (*n* = 27) and *L. alleni* (*n* = 30). (**A**) Mass versus SVL. (**B**) Maximal gape area versus SVL. (**C**) Maximal gape area versus mass.

For a given SVL, both the mass (Fig. 2A) and maximal gape area (Fig. 2B) of *L. alleni* were significantly greater than those of *R. septemvittata*, whereas, for a given mass, the gape of *R. septemvittata* was significantly larger than that of *L. alleni* (Fig. 2C). Supplementary Table S1 provides some predicted values and 95% confidence limits of maximal gape areas to clarify the magnitude of differences between species that are difficult to discern in the log–log plots (Fig. 2). In both species, maximal gape scaled with negative allometry relative to SVL (diameter slope < 1; area slope < 2) and mass (diameter slope < 0.33; area slope < 0.67) (Table 1). The only significant differences (both *P *>* *0.025) between sexes within a species were for gape diameter and area as a function of mass for *R*. *septemvittata*. The soft tissue between the lower jaws of both species accounted for approximately one-sixth of the maximal gape area (Table 2 and Fig. 1), which emphasizes the importance of measuring gape directly rather than estimating it for skeletal dimensions. The cranial elements of both species had similar contributions to gape area except for the quadrate bones, which contributed significantly more in *L. alleni* than in *R. septemvittata* (Table 2).

**Table 1:** Regression analyses of morphology and prey HTs

Dependent variable	Independent variable	Slope ± 95% CL	Intercept ± 95% CL	*n*	*R* ^2^
*R. septemvittata*					
log Mass (g)	log SVL (mm)	2.930 ± 0.127	−6.156 ± 0.314	27	0.99
log Diam (mm)	log SVL (mm)	0.815 ± 0.054	−0.874 ± 0.133	27	0.97
log Area (mm^2^)	log SVL (mm)	1.630 ± 0.108	−1.854 ± 0.266	27	0.97
log Diam (mm)	log Mass (g)	0.274 ± 0.024	0.843 ± 0.028	27	0.96
log Area (mm^2^)	log Mass (g)	0.548 ± 0.048	1.581 ± 0.057	27	0.96
log HT (s)	log RPA (%) (all)	1.329 ± 0.211	0.444 ± 0.306	118	0.57
log HT (s)	log RPA (%) (soft)	1.689 ± 0.293	−0.239 ± 0.436	38	0.79
log HT (s)	log RPA (%) (medium)	1.134 ± 0.257	0.795 ± 0.368	80	0.49
log HT (s)	log RPM (%) (all)	0.826 ± 0.135	1.629 ± 0.129	118	0.55
log HT (s)	log RPM (%) (soft)	1.080 ± 0.184	1.238 ± 0.188	38	0.79
log HT (s)	log RPM (%) (medium)	0.692 ± 0.165	1.817 ± 0.151	80	0.46
*L. alleni*					
log Mass (g)	log SVL (mm)	3.007 ± 0.150	−5.970 ± 0.354	30	0.98
log Diam (mm)	log SVL (mm)	0.796 ± 0.045	−0.758 ± 0.106	30	0.98
log Area (mm^2^)	log SVL (mm)	1.593 ± 0.090	−1.620 ± 0.212	30	0.98
log Diam (mm)	log Mass (g)	0.261 ± 0.018	0.827 ± 0.022	30	0.97
log Area (mm^2^)	log Mass (g)	0.522 ± 0.036	1.549 ± 0.044	30	0.97
log HT (s)	log RPA (%) (all)	1.262 ± 0.237	0.480 ± 0.364	127	0.47
log HT (s)	log RPA (%) (molted)	1.676 ± 0.297	−0.339 ± 0.479	66	0.66
log HT (s)	log RPA (%) (hard)	1.501 ± 0.296	0.308 ± 0.429	61	0.63
log HT (s)	log RPM (%) (all)	0.546 ± 0.142	1.984 ± 0.123	127	0.31
log HT (s)	log RPM (%) (molted)	0.719 ± 0.190	1.708 ± 0.182	66	0.46
log HT (s)	log RPM (%) (hard)	0.581 ± 0.203	2.094 ± 0.154	61	0.35

All regressions had *P *<* *0.001. Prey types in analyses for *L. alleni* log HT were: all = soft, medium, and hard-shelled prey, molted = soft-shelled and medium prey hardness, and hard = hard-shelled prey only.

**Table 2:** Mean percent contributions to maximal gape area and comparisons between species

	Mean values ± SE			
Contribution	*R. septemvittata*	*L. alleni*	*t*	*P*
Skull width	13.9 ± 0.3	13.4 ± 0.4	1.122	0.277
Quadrate	11.3 ± 0.4	14.2 ± 0.5	−4.918	<0.001
Lower jaw	57.9 ± 1.3	57.5 ± 1.5	0.227	0.823
Skin and other intermandibular soft tissues	16.9 ± 1.1	15.0 ± 1.6	0.994	0.334

Measured from CT scans (Fig. 1) of individuals with SVL from 163 to 586 mm and 140 to 473 mm for *R. septemvittata* (*n *=* *10) and *L. alleni* (*n *=* *9), respectively.

### Diet and prey size

**Fig. 3:**
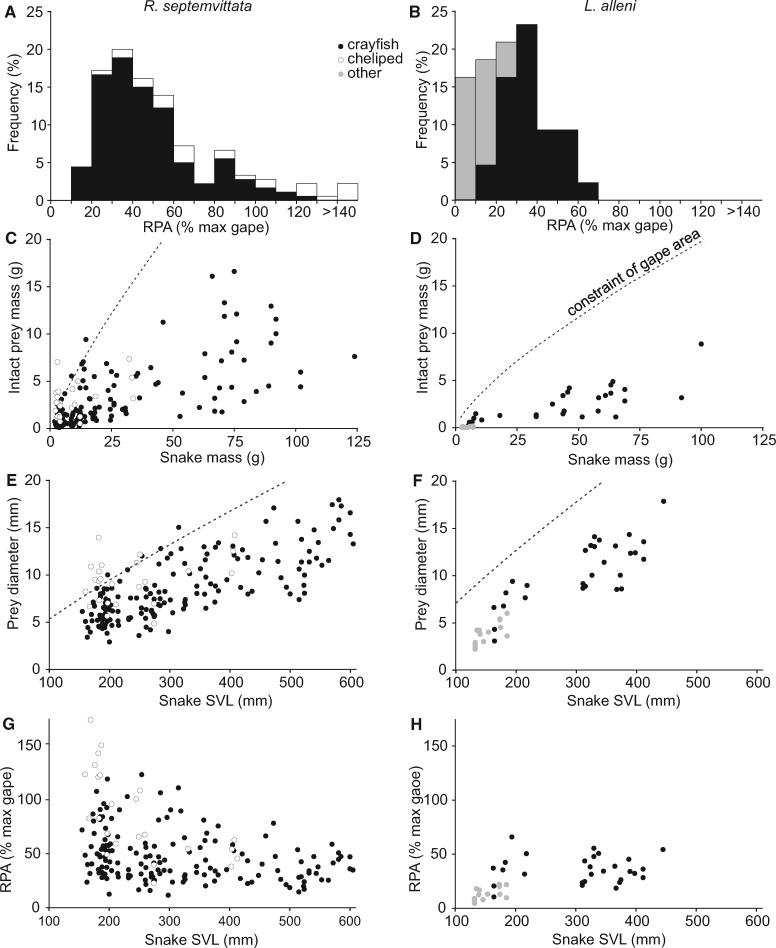
Absolute and relative sizes of prey consumed in the field by *R. septemvittata* (left; *n* = 180) and *L. alleni* (right; *n* = 43). For the 25 chelipeds consumed by *R. septemvittata*, values of relative prey size were calculated based on the predicted size of an intact crayfish from scaling equations for that species. With the exception of 17 *L. alleni*, for which gape was measured directly, all other values of gape were estimated by using the SVL of the snake and the scaling equations in Table 1. (**A**, **B**) Frequency distributions of consumed prey based on cross-sectional area of the prey relative to the maximal gape area of the snake (RPA). The crayfish consumed by *R. septemvittata* and *L. alleni* were *O. rusticus* and *P. fallax*, respectively. Other prey consumed by *L. alleni* included 15 odonate nymphs (*M. marcella*), and 1 grass shrimp (*P. paludosus*). (**C**, **D**) Prey mass versus snake mass. The dashed lines indicate predicted the mass of prey when RPA is 100% based on the size of the snake that consumed it. (**E**, **F**) Prey maximal diameter versus SVL of the snake. The dashed lines indicate the predicted diameter of prey when it is 100% of the gape diameter based on the SVL of the snake that consumed it. (**G**, **H**) RPA versus SVL of the snake.

In 55% of 340 captured *R. septemvittata*, we recovered parts from 194 freshly molted crayfish (*O. rusticus*), and of these stomach contents 25 were only a cheliped. For all the whole crayfish consumed by *R. septemvittata*, 63% were swallowed tail first, and all of the detached chelipeds were ingested from distal to proximal. For the crayfish that were consumed whole, we were able to estimate the intact size, RPA, and RPM, for 155 crayfish. Values of RPA of crayfish consumed whole ranged from 11% to 123% (mean = 47.2 ± 1.88%) (Fig. 3A), and RPM ranged from 2% to 85% (mean = 17.4 ± 1.2%). For the 25 chelipeds, the values of RPA and RPM ranged from 3% to 81% (mean = 25.0 ± 3.4%) and from 0.3% to 37% (mean = 7.1 ± 1.6%), respectively. The estimated sizes of the intact crayfish for these chelipeds had RPA ranging from 23% to 226% (mean = 91.4 ± 9.7%) and RPM ranging from 4% to 236% (mean = 67.8 ± 14.5%). In addition to some *R*. *septemvittata* removing chelipeds from crayfish with RPA > 100%, six of the intact crayfish consumed had RPA > 100%. Of these six crayfish, three had values of RPA (103–107%) that seem readily explained by errors in predicting gape area from SVL (Supplementary Table S1). Perhaps the remaining three crayfish with RPA of 111–123% were soft enough that the snakes could deform them during swallowing. As SVL increased, the ranges in absolute prey diameters were quite similar (Fig. 3E), whereas smaller snakes had larger ranges of RPA and were more likely to consume relatively large prey (Fig. 3G).

Of the 35 *L. alleni* that we collected plus the 68 *L. alleni* collected by Godley (1980), 44 snakes (43%) contained 54 identifiable prey items consisting of 55.6% crayfish, (*P. fallax*) (1 recently molted; 29 hard-shelled), 42.6% odonate nymphs (*Miathyria marcella*), and 1.9% grass shrimp (*Palaemonetes paludosus*), and we estimated intact size for 43 of these items (62.8% crayfish, 34.9% odonates, and 2.3% shrimp) from 33 snakes. All crayfish were consumed whole and tail first, and their values of RPA and RPM ranged from 10% to 66% (mean = 35.7 ± 2.4%) and 1.3% to 18% (mean = 6 ± 0.7%), respectively (Fig. 3B). Values of RPA and RPM of the odonate nymphs ranged from 4% to 22% (mean = 12 ± 1.5%) and 0.3% to 3.4% (mean = 1.4 ± 0.3%), respectively. The one grass shrimp had RPA = 17% and RPM = 0.3%. Hence, most of the prey with RPA < 20% were consumed by small snakes and were odonates (Fig. 3H).

The frequency distributions of RPA for whole crayfish did not differ significantly between *R. septemvittata* (*n *=* *155) and *L. alleni* (*n *=* *28) (Fig. 3A, B; *D *=* *0.21, *P *=* *0.227), or when the estimated intact sizes of the entire crayfish were included for when only chelipeds were consumed by *R*. *septemvittata* (*n *=* *180) (*D* = 0.26, *P *=* *0.072). However, the frequency distributions of RPA for the combined samples of all whole prey consumed by *L. alleni* (*n *=* *43) and *R*. *septemvittata* (*n *=* *155) had highly significant differences (*D *=* *0.33, *P *=* *0.002) as a result of *L. alleni* consuming more small prey (Fig. 3B).

### Feeding behavior and HT

**Fig. 4:**
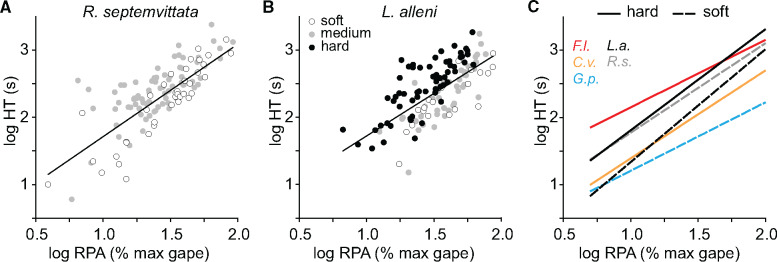
Prey HTs versus RPA for crustacean-eating snakes. Regressions for the entire sample of intact crayfish consumed by (**A**) *R. septemvittata* (*n* = 118) and (**B**) *L. alleni* (*n* = 127) in the laboratory. (**C**) Regressions of HT versus RPA performed separately based on molt status (solid versus dashed lines) for: R.s., *R. septemvittata* eating *O. rusticus*; L.a., *L. alleni* eating *P. fallax*; F.l., *Fordonia leucobalia* eating crabs swallowed side to side; G.p., *Gerarda prevostiana* eating crabs swallowed front to back; C.v., *Cantoria violacea* eating snapping shrimp. See Table 1 for regression statistics for the crayfish-eating species. The homalopsid data are from Jayne et al. (2018).

We fed 8 *R. septemvittata* 121 freshly molted crayfish (*O. rusticus*) with RPA (Fig. 4A) and RPM ranging 4–154% and 1–124%, respectively. *Regina septemvittata* usually struck the carapace of the crayfish, and 26% of the trials required more than one strike to capture the crayfish (Table 3). Tail flips allowed the crayfish to escape strikes at least once in 27% of the trials. Jaw holding behavior occurred in 82% of the trials. In 36% of the trials during which the crayfish flipped their tails after being captured, they escaped. In 61% of the trials, the crayfish were swallowed with their lateral surface contacting the palate of the snake.

**Table 3:** Summary of prey handling behaviors during laboratory trials

Behavior	Percent of trials with behavior	
	***R. septemvittata* (*n *=* *118)**	***L. alleni* (*n *=* *127)**
Crayfish		
Pre-capture tail flip + escape	27^R^	13^R^
Pre-capture pinching	5	0
Post-capture tail flip	36^R,H^	46^R,H^
Post-capture tail flip + escape	12	4^R,H^
Post-capture pinch	19^H^	48^H^
Post-capture pinch + escape	2	1
Snake		
Cheliped removal	16^a^	0
>1 unsuccessful strike	26^R,H^	13
Strike location		
Cheliped	2	0
Carapace	49	27
Carapace-abdomen joint	18	35
Abdomen	31	38^R^
Hold prey before swallowing	82^R,H^	11
Body restraint	0	69
U-loop	0	18^b^
Pin	0	28^b^
Coil	0	43^b^
Crayfish orientation at swallow		
Dorsal	24	1
Lateral	61	90
Ventral	14	9
Direction swallow		
Head	22	2
Mid-body	3	1
Tail	73^H^	97^R^
Chela	3	0

R and H indicate that when the presence (1) or absence (0) of a behavior in the left column was a dependent variable in a univariate regression, it changed significantly with increased RPA and prey hardness, respectively (Supplementary Table S4). ^a^Total *n *=* *121 including three trials in which only the cheliped was removed; ^b^More than one type of body restraint behavior could occur within a single trial.

In 19 trials *R*. *septemvittata* removed and ate a cheliped, and these chelipeds were always swallowed from distal to proximal. In three of these cases, snakes only consumed the cheliped (whole crayfish RPA = 26%, 65%, and 154%), whereas in 16 cases after eating a cheliped, the snake subsequently consumed the entire crayfish (RPA = 7–64%).

Prey HTs of *R*. *septemvittata* ranged from 6 to 2376 s and increased significantly in univariate regressions with increased values of RPA (*R*^2^ = 0.57, Fig. 4A) or RPM (*R*^2^ = 0.55) (Table 1). For the 118 feedings when the entire crayfish was consumed, a multiple regression revealed that HT increased significantly (overall *R*^2^ = 0.66, *P *<* *0.001) with increases in RPA (*P *<* *0.001), prey hardness (*P *<* *0.001), strike location (*P *=* *0.007), and the number of crayfish pinches (*P *=* *0.025) (Supplementary Table S3). Another regression revealed that the presence of the jaw holding behavior (1 = present, 0 = absent) was significantly more likely to occur (overall *R*^2^ = 0.42, *P *<* *0.001) with increases in RPA (*P *<* *0.001), prey hardness (*P *<* *0.001), and strike location (*P* = 0.007) (Supplementary Table S3).

We fed 15 *L. alleni* 66 freshly molted (RPA = 13–83%, RPM = 1–36%) and 61 hard-shelled (RPA = 7–62%, RPM = 1–23%) *P. fallax* (Fig. 4B and  Table 3). The final strike hit the abdomen, carapace, and the carapace–abdomen joint in 38%, 27%, and 35% of the trials, respectively. In 13% of the trials more than one strike was required to capture the crayfish. Less than 10% of the post-capture tail flips allowed the crayfish to escape. We never observed *L. alleni* striking or removing a cheliped. The snakes displayed jaw holding in only 11% of the trials. Snakes used their body to restrain prey in 69% of the trials. In 97% and 90% of the trials, prey were swallowed tail first and with their lateral surface contacting the palate, respectively.

**Fig. 5:**
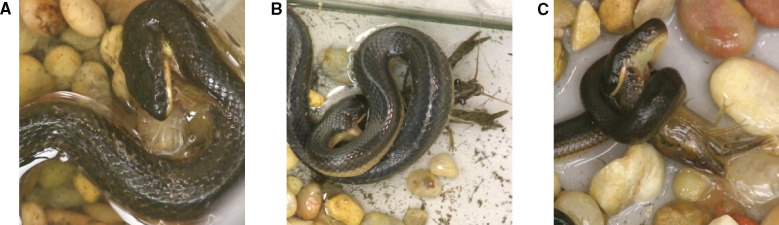
Prey restraint behaviors of *L. alleni* attacking crayfish. (**A**) U-loop of a snake (SVL = 440 mm, [gravid] mass = 142 g) with a soft-shelled crayfish (RPA = 24%, mass = 2.5 g). (**B**) Pinning by a snake (SVL = 326 mm, mass = 44 g) with a hard-shelled crayfish (RPA = 56%, mass = 4.0 g). (**C**) Coiling by a snake (SVL = 187 mm, mass = 5.5 g) with a soft-shelled crayfish (RPA = 80%, mass = 1.7 g).

*Liodytes alleni* used the following categories of increasingly greater amounts of body restraint: (0) no body restraint, (1) U-loop during which the snake pushed the crayfish against a concave side of its body that partially encircled crayfish (Fig. 5A), (2) body pinning during which the ventral surface of the snake pushed down on the crayfish (Fig. 5B), or (3) coiling, during which the snake completely encircled the crayfish (Fig. 5C). RPA better predicted the amount of prey restraint for hard-shelled (*n *=* *61, *R*^2^ = 0.19, *P *<* *0.001) than for soft-shelled crayfish (*n *=* *66, *R*^2^ = 0.09, *P *=* *0.013) (Supplementary Table S4). For the combined sample of soft- and hard-shelled crayfish, *L. alleni* used increasingly greater categories of body restraint with increased RPA (*n *=* *127, *R*^2^ = 0.06, *P *=* *0.005), but more than three times as much variance (overall *R*^2^ = 0.21, *P *<* *0.001) in the categories of body restraint was accounted for in a multiple regression that included RPA (*P *<* *0.001), prey hardness (*P *<* *0.001), and the number of pinches (*P *=* *0.02) as independent variables (Supplementary Table S3).

In univariate regressions performed separately for hard- and soft-shelled crayfish consumed by *L*. *alleni* (Table 1), HT increased significantly both with increased RPA (Fig. 4B) and RPM, but the *R*^2^ for RPA exceeded that for RPM. In a multiple regression, the HTs pooled for hard- and soft-shelled crayfish increased significantly (*n *=* *127, *R*^2^ = 0.71, *P *<* *0.001) with increases in RPA (*P *<* *0.001), body restraint of prey (*P *=* *0.001), prey hardness (*P *<* *0.001), and number of pinches after capture (*P *=* *0.007) (Supplementary Table S3).

In addition to molt status affecting HTs, it also affected maximal sizes of prey consumed and attacked. In captivity the 13 *L. alleni* that consumed both hard- and soft-shelled crayfish had significantly greater maximal sizes for soft- versus hard-shelled crayfish that were successfully consumed (paired *t *=* *6.29, *P *<* *0.001) and for soft- versus hard-shelled crayfish that were attacked but not consumed (paired *t *=* *4.37, *P *<* *0.001).

Crayfish attacked by both species of snakes displayed the following defensive behaviors: (1) raising the chelipeds to fend off the snake, (2) using a tail flip to flee from the snake, or (3) pinching the snake (Supplementary Video). After capture, however, pinching rarely resulted in an escape.

## Discussion

### Convergent evolution of dietary specialists

The ability of predators to exploit prey is affected by attributes of the prey such as rarity, absolute size, mobility, strength, defensive behaviors, and resistance to physical injury and death and attributes of the predator such as the size and the abilities to detect, subdue, consume, and digest prey (Endler 1991; Arnold 1993). Many snake species with generalized dentition and no conspicuous specializations in behavior eat fishes and amphibians, and based on recent phylogenies (Figueroa et al. 2016), this is the most parsimoniously inferred diet of the most recent common ancestors of the crayfish-eating natricines (*Liodytes*, *Regina*) and the crustacean-eating homalopsids (*Cantoria*, *Fordonia*, *Gerarda*) (Gibbons and Dorcas 2004; Jayne et al. 2018). Recent phylogenies (Figueroa et al. 2016) also imply that within natricines the specialized diet of primarily crayfish evolved independently three times, and the diet of soft-shelled crayfish evolved twice. The two crab-eating homalopsid genera (*Fordonia* [hard-shelled]; *Gerarda* [soft-shelled]) are monophyletic, and their sister taxon, *Cantoria*, eats hard-shelled snapping shrimp and crabs (Ghodke et al. 2018; Jayne et al. 2018). Hence, in both natricines and homalopsids no evidence suggests that the diet of more readily consumed soft-shelled crustaceans preceded a diet of mostly hard-shelled crustaceans.

Several similar and convergent trends occur within natricines and homalopsids that eat hard- versus soft-shelled crustaceans. Compared with those that prey on soft-shelled crustaceans, the genera that eat hard-shelled prey: (1) have blunter teeth, (2) thickened stomachs, (3) larger gape relative to SVL, (4) larger mass relative to SVL, and (5) consume prey with smaller values of RPA and RPM (Savitzky 1983; Jayne et al. 2018).

The use of the body to restrain or constrict prey has evolved repeatedly within snakes (Greene and Burghardt 1978), and this behavioral specialization seems likely to facilitate handling prey that are large or difficult to subdue. However, using the body to restrain prey is uncommon in both natricines (Gregory et al. 1980) and homalopsids (Jayne et al. 2018). The two species of *Liodytes* that eat hard-shelled crayfish and use body restraint (Tumlison and Roberts 2018) are a monophyletic group (Figueroa et al. 2016). All homalopsid species that are crustacean specialists also use body restraint (Jayne et al. 2018) and are a monophyletic group (Figueroa et al. 2016). Because none of the sister taxa to either of these two clades use body restraint (Gibbons and Dorcas 2004; Jayne et al. 2018), the most parsimonious explanation for both clades is that body restraint evolved independently in the most recent common ancestor of each of these clades. With increased RPA, the extent of encircling prey with the body of *L*. *alleni* (Fig. 5) increases significantly as does the amount of body restraint used by *Gerarda* (Jayne et al. 2018). However, *Fordonia* is more stereotyped in that it uses its body to restrain crabs of nearly all sizes, but the body postures that are used can be quite variable (Jayne et al. 2018). Compared with *R*. *septemvittata*, which does not use body restraint, the faster prey handling (Fig. 4C) and reduced percentage of trials with escapes in *L*. *alleni* (Table 3) provide compelling support for the benefits of snakes using their body to restrain prey.

Another specialized and very rare behavior in snakes is consuming prey in pieces, and doing so circumvents the limitations of gape on the size of prey that can be exploited (Jayne et al. 2018). We observed this in both laboratory trials and for the prey consumed in the field by *R*. *septemvittata*, and this was significantly more likely to occur for snakes in the field as RPA increased (*n *=* *180, *R*^2^ = 0.17, *P *<* *0.001). *Cantoria*, *Fordonia*, and *Gerarda* also all break off appendages of their crustacean prey (Ghodke et al. 2018; Jayne et al. 2018). *Gerarda* also combines body restraint with ripping apart the carapace of crabs to eat crabs that are too large to be swallowed whole, and *Gerarda* has remarkably fast prey HTs for a given RPA compared with all of the other crustacean-eating species (Fig. 4C). Thus, consuming pieces of prey (usually appendages) is reasonably common in snakes that specialize in crustaceans. Furthermore, the specialized behaviors of *R*. *septemvittata* and *G. prevostiana* allow them to exploit prey with intact sizes that are too large to be consumed whole.

Given the formidable body plans of crustaceans, why would snakes evolve this prey preference? Perhaps, the primary advantage of crustaceans as prey is their abundance. The habitats of *L*. *alleni* can have extremely high densities of crayfish (61 m^−2^) (Godley 1980), and in mangroves where crustacean-eating homalopsids are found, the biomass of crabs may be as high as 80% of the entire macrofauna with densities as high as 80–90 m^−2^ (Macintosh 1984, 1988). Despite the high abundance of these crustaceans, their energetic content per unit wet mass (Godley 1980; Du Preez and McLachlan 1983) is lower than that of some fish and amphibians consumed by natricine snakes (Willson and Hopkins 2011). However, the energetic content of crustaceans can increase after molting (Godley 1980), and both the natricine and homalopsid snake species that exploit this prey resource are adept at using olfactory cues to distinguish freshly molted from hard-shelled individuals (Jackrel and Reinert 2011; Jayne et al. 2018).

### Effects of predator and prey size on feeding performance

An advantage of studying the foraging ecology of snakes is that identifying prey species and quantifying their size is facilitated by a lack of mastication, and the maximal gape of snakes usually limits maximal prey size. Patterns of predator and prey size within a species of snake have been analyzed most commonly by plotting a single measure of prey size versus a single measure of snake size (as in Fig. 3C–F). As reviewed in Arnold (1993), two recurrent findings from such bivariate plots are with increased snake size: (1) the mean prey size increases and (2) the variance in prey size increases. In addition, larger snakes of many species often do not continue to eat small prey; hence, the cloud of points has a wedge-like shape. By contrast, such data for a lesser number of species form a right triangle with an upward sloping hypotenuse forming the upper boundary. What determines the lower limit of prey size in snakes remains unclear (Arnold 1993), but behavior may be one factor. The upper boundary is constrained by the maximal gape of snakes, but if snakes simply choose not to consume large prey, then the upper boundary could be well below that imposed by the constraint of gape. However, no previous study has directly measured gape and integrated this with plots of prey and snake size.

We observed substantially different patterns of prey and snake size depending on the metric of size. The data for prey mass versus snake mass resembled a right triangle (Fig. 3C, D), whereas those for prey diameter versus SVL resembled a parallelogram with unexpectedly similar ranges in prey diameter over a wide range in SVL (Fig. 3E, F). Values of RPA versus SVL, especially for *R*. *septemvittata*, resembled a right triangle with a downward sloping hypotenuse (Fig. 3G). However, *L*. *alleni* (Fig. 3H) lacked this conspicuous contraction in RPA with increased SVL. At least for our study species, fitting a line through the upper points of the data for prey size versus snake size as in (King 2002) would underestimate maximal gape.

All metrics of prey and snake size indicate that *R*. *septemvittata* consumed prey that were larger relative to their size more commonly than *L*. *alleni*, but this difference between species was most pronounced for the juveniles (Fig. 3). The timing of the reproduction of the crayfish and snakes causes a seasonal scarcity of small crayfish for the young of the year for both *R*. *septemvittata* (Prins 1968) and *L*. *alleni* (Godley 1980), but these two species cope with this in different ways. Besides eating whole crayfish with larger relative size, *R. septemvittata* eats chelipeds from crayfish larger than can be swallowed whole, whereas *L*. *alleni* eats many small odonate nymphs (Fig. 3H). Hence, many young *R*. *septemvittata* could be viewed as “overachievers” or “olympians” that use a high fraction of their maximal capacity (Hertz et al. 1988). This resembles other systems where animals in the field compensate for an inferior maximal capacity by using a larger proportion of their maximal capacity (Husak and Fox 2006).

**Fig. 6:**
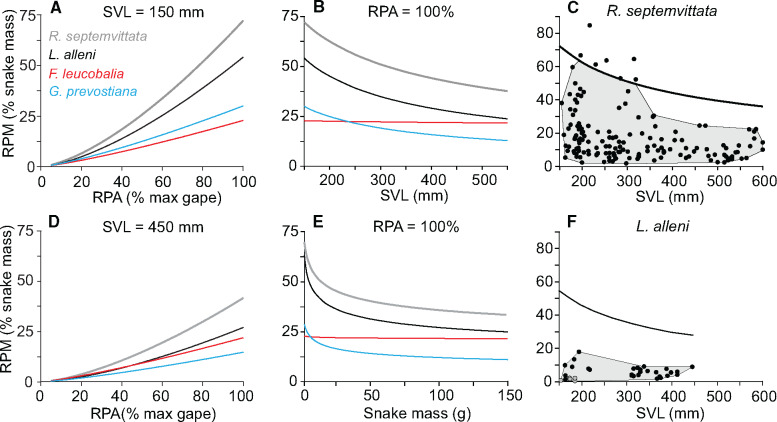
Effects of maximal gape and snake size on relative mass of prey (RPM). In all panels (**A**–**F**) the areas below the curves represent the potential feeding performance space available to the snakes. In panels C and F, symbols show the sizes of whole prey consumed in the field and the gray areas indicate the realized feeding performance spaces.

One good choice of currency for understanding foraging ecology is the mass of the prey relative to that of the predator because the former is proportional to the energy consumed and the latter is proportional to the energetic need. Thus, RPM has long been used to quantify prey size for hundreds of species of snakes (Voris and Moffett 1981; Glaudas et al. 2019). However, RPM is not sufficient to determine the size of prey consumed relative to the maximal size that is possible. Hence, we integrated our scaling data and direct measurements of snake gape to define various performance spaces that included both RPA and RPM (Fig. 6).

For the natural prey of four species of crustacean-eating snakes, we plotted RPM versus RPA to gain insights into the benefits for RPM from eating prey with different RPA (Fig. 6A, D). The three species (*R*. *septemvittata*, *L*. *alleni*, and *G*. *prevostiana*) with negative allometry of gape area and SVL (slope < 1.67) had the following two trends. First, for a given RPA and species, RPM decreases with increased snake size (Fig. 6A versus Fig. 6D). Second, a nonlinear relationship causes the benefits in RPM to increase with increased RPA (Fig. 6A, D). For example for a *R*. *septemvittata* with SVL = 150 mm, as RPA increases from 20% to 30%, and from 80% to 90%, RPM increases from 7% to 12% and 52% to 62%, respectively. In addition, for a given RPA, the crayfish-eating species can consume prey with larger RPM than the two crab-eating species (Fig. 6A, D). As a result of positive allometry between gape area and SVL for *F*. *leucobalia*, the relationship between RPM and RPA was nearly unaffected by SVL (Fig. 6A versus Fig. 6D).

We also used the areas under the curves of RPM versus overall snake size (when prey cross-sectional area equals maximal gape area) to define potential performance spaces based on the constraints of gape on prey size (Fig. 6B, E). When either SVL or mass was used for overall snake size, over nearly the entire range of snake size the rank order from greatest to smallest values of RPM was *R*. *septemvittata*, *L*. *alleni*, *F*. *leucobalia*, and *G*. *prevostiana* (Fig. 6B, E). Consequently, the areas under these curves differed substantially (Fig. 6C, for SVL = 150–550 mm) as values for *L*. *alleni*, *F*. *leucobalia*, and *G*. *prevostiana* were 68%, 44%, and 37%, respectively, of that for *R*. *septemvittata*. The realized performance spaces of RPM versus SVL for *R*. *septemvittata* and *L*. *alleni* were approximately 75% and 26% of the potential performance space, respectively (Fig. 6C, F). Hence, *L*. *alleni* had a smaller potential feeding performance space and unexpectedly used less of it than *R*. *septemvittata*.

**Fig. 7:**
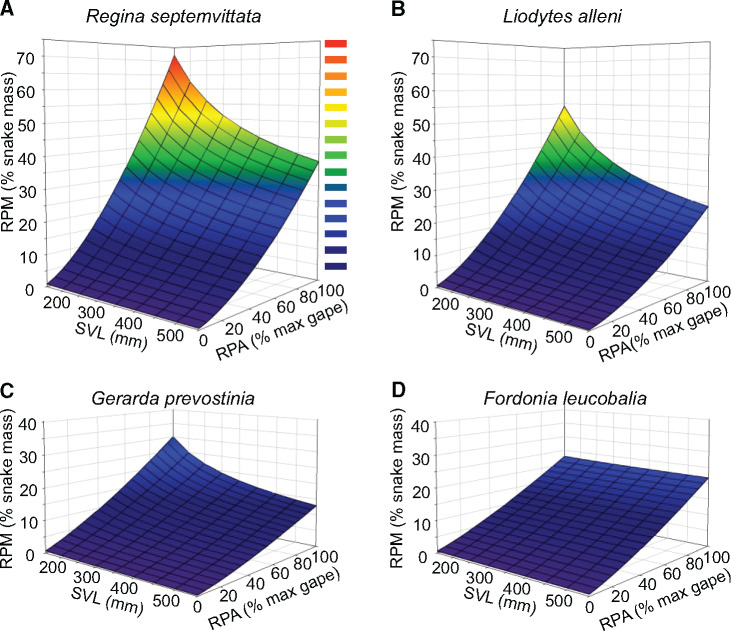
Three-dimensional feeding performance spaces delineated by RPM as a function of SVL and RPA for four crustacean-eating specialists consuming their normal prey (as in Fig. 4). Homalopsid data are from Jayne et al. (2018). Overall, crayfish-eating snakes have larger performance spaces than crab-eating snakes, and juvenile snakes can usually consume prey with larger RPM for a given RPA.

One can also delineate a performance space based on the volume beneath a three-dimensional surface defined by RPM as a function of SVL and RPA (Fig. 7). For a range of SVL in common to the four crustacean-eating species (150–550 mm), the rank order of species based on their feeding performance volumes was the same as those based on the areas defined by curves of RPM versus snake size when RPA equaled 100% (Fig. 6B, E).

Most of the prey consumed by our study species were much smaller than the limit imposed by gape (Fig. 6C, F), and this was especially true for *L*. *alleni*. Similarly, in the field *F*. *leucobalia* consumes hard-shelled crabs with small RPA (Jayne et al. 2018). Thus, the foraging strategies of *L*. *alleni* and *F*. *leucobalia* run counter to the intuitive expectation that species with a small performance space might use a greater faction of it to accrue benefits for increased RPM. By contrast, *G*. *prevostiana* has a very small performance space (Fig. 6B, E), and it does conform to this expectation as it often tears apart whole prey larger than its maximal gape and some of these pieces actually approach the limits of its gape (Jayne et al. 2018).

For the species that eat hard-shelled crustaceans, the risk of injury is significant and increases with increased prey size (Godley 1980). Laboratory observations combined with the small RPA of prey consumed in the field also suggest that successful capture is difficult. Furthermore, although snakes in cages may recapture crayfish after they escape, this seems much less likely in the field. Hence, we propose that the difficulty of prey capture and handling are major factors contributing to the rarity of hard-shelled crustacean prey with large RPA for wild snakes. The snakes also may simply choose to not attack prey that are large and formidable. Nevertheless, these factors emphasize the extremely important roles of behavior in determining the patterns of resource use that occur even after accounting for the constraints imposed by anatomy.

One could use similar approaches to those described herein to provide further insights into the potential effects of consuming species of prey that differ in shape as well as size. Theoretically, for a given gape area, gape-limited predators could attain the same values of RPM from eating either stout prey or elongate prey that have much smaller RPA. Indeed, many elongate species of prey are consumed by snakes such as eels, salamanders, and snakes (Voris and Voris 1983; Jackson et al. 2004; Willson and Hopkins 2011; Sherratt et al. 2019). However, some species of sea snakes that specialize on eels and elongate fishes have such small heads that it seems likely that their gape is small (Voris and Voris 1983; Sherratt et al. 2019). Consuming elongate prey could allow some species to exploit prey with high RPM by using only a small fraction of their maximal gape capacity (Greene 1983), but some species with small heads and likely small gape may actually consume prey that are elongate and have high values of RPA. Direct measurements of gape and prey size could resolve these interesting possibilities.

Many species of prey consumed by snakes including crayfish have nearly circular cross-sectional areas, but prey such as laterally compressed species of fish depart substantially from this shape (Willson and Hopkins 2011). Close relatives of the crayfish-eating snakes in the genus *Nerodia* have relatively large heads, and their diet includes laterally compressed species of fishes such as the genus *Lepomis* (Mushinsky et al. 1982; Willson and Hopkins 2011). The larger heads of *Nerodia* seem likely to have larger gape, which could facilitate consuming prey either with large nearly circular cross-sectional areas or with modest cross-sectional areas but large ratios of height to width. Perhaps, having large gape relative to prey cross-sectional area could also facilitate consuming prey that have irregular shapes (Vincent et al. 2006) or are difficult to handle such as hard-shelled crayfish and hard-shelled crabs. However, many groups of snakes including *Liodytes* (Gibbons and Dorcas 2004) and the crustacean-eating homalopsids burrow (Jayne et al. 2018), and the microcephalic sea snakes probe narrow spaces when they forage (Voris and Voris 1983). Having small heads to facilitate these tasks may thus create tradeoffs with the morphologies that enhance gape (Fabre et al. 2016) and facilitate consuming non-circular prey.

Size and anatomy have profound effects on all aspects of animal biology including ecology. Our study illustrates how using functional morphology is a powerful first step for defining what is possible (maximal performance), and our field data tested the fraction of maximal performance that was realized in nature. Laboratory observations illuminated the importance of behavior for determining the success and difficulties in predator–prey interactions. Despite the small number of snake species for which gape has been measured directly, some strikingly different foraging strategies are already becoming apparent. For example, we identified convergent patterns in two taxa (*Regina* and *Gerarda*) that consume relatively large and helpless prey such as recently molted crustaceans, whereas two others (*Liodytes* and *Fordonia*) eat formidable prey (hard-shelled crustaceans) with smaller sizes relative to their gape. Relative prey size predicted the largest fraction of the variance in prey HTs, but prey hardness, prey defensive behaviors, and the use of body restraint by the snakes also affected HTs and the success of the predator. Similar future integrative studies expanding the comparative data that are available should facilitate understanding the relative importance of anatomy, behavior, and phylogeny for large scale patterns in variation in foraging ecology.

## Supplementary Material

obab001_Supplementary_Data
