# Supplementary material for: Crayfish Eating in Snakes: Testing How Anatomy and Behavior Affect Prey Size and Feeding Performance
Source: Integr Org Biol. 2021 Jan 30;3(1):obab001. doi: 10.1093/iob/obab001 (PMC8023418; doi:10.1093/iob/obab001)
Supplement: obab001_Supplementary_Data [file obab001_supplementary_data.zip › IOB-2020-039.R2_Fig_Captions.docx]

Figure Captions

**Fig. 1** Contributions of skeletal elements and soft tissues to maximal gape area**.** Anterior views of computed tomography scans of *R. septemvittata* (**A**) and *L. alleni* (**B**) preserved at maximum gape. The relative contributions to maximal gape area are shown for the skull width (SW), quadrate (QD), lower jaw (LJ), and the skin and intermandibular ligament between the lower jaws (SI). See Table 2 for mean values and comparisons between species.

**Fig. 2** Scaling relationships for morphological data of *R. septemvittata* (*n* = 27) and *L. alleni* (*n* = 30)*.* (**A**) Mass versus snout-vent length. (**B**) Maximal gape area versus snout-vent length. (**C**) Maximal gape area versus mass.

**Fig. 3** Absolute and relative sizes of prey consumed in the field by *R. septemvittata* (left; *n* = 180) and *L. alleni* (right; *n* = 43). For the 25 chelipeds consumed by *R*. *septemvittata*, values of relative prey size were calculated based on the predicted size of an intact crayfish from scaling equations for that species. With the exception of 17 *L*. *alleni*, for which gape was measured directly, all other values of gape were estimated by using the SVL of the snake and the scaling equations in Table 1. (**A**, **B**) Frequency distributions of consumed prey based on cross-sectional area of the prey relative to the maximal gape area of the snake (RPA). The crayfish consumed by *R*. *septemvittata* and *L*. *alleni* were *Orconectes* *rusticus* and *Procambarus fallax*, respectively. Other prey consumed by *L*. *alleni* included 15 odonate nymphs (*Miathyria marcella*), and 1 grass shrimp (*Palaemonetes* *paludosus*). (**C**, **D**) Prey mass versus snake mass. The dashed lines indicate predicted the mass of prey when RPA is 100% based on the size of the snake that consumed it. (**E, F**) Prey maximal diameter versus SVL of the snake. The dashed lines indicate the predicted diameter of prey when it is 100% of the gape diameter based on the SVL of the snake that consumed it. (**G, H**) Relative prey area versus SVL of the snake.

**Fig. 4** Prey handling times versus RPA for crustacean-eating snakes. Regressions for the entire sample of intact crayfish consumed by (**A**) *Regina septemvittata* (*n* = 118) and (**B**) *Liodytes alleni* (*n* = 127) in the laboratory. (**C**) Regressions of handling time versus RPA performed separately based on molt status (solid versus dashed lines) for: *R.s*., *R. septemvittata* eating *Orconectes rusticus*; *L.a*., *L. alleni* eating *Procambarus fallax*; *F.l*., *Fordonia leucobalia* eating crabs swallowed side to side; *G.p*., *Gerarda* *prevostiana* eating crabs swallowed front to back; *C.v*., *Cantoria violacea* eating snapping shrimp. See Table 1 for regression statistics for the crayfish-eating species. The homalopsid data are from (Jayne et al. 2018).

**Fig. 5** Prey restraint behaviors of *Liodytes alleni* attacking crayfish. (**A**) U-loop of a snake (SVL = 440 mm, [gravid] mass = 142 g) with a soft-shelled crayfish (RPA = 24%, mass = 2.5 g). (**B**) Pinning by a snake (SVL = 326 mm, mass = 44 g) with a hard-shelled crayfish (RPA = 56%, mass = 4.0 g). (**C**) Coiling by a snake (SVL = 187 mm, mass = 5.5 g) with a soft-shelled crayfish (RPA = 80%, mass = 1.7 g).

**Fig. 6** Effects of maximal gape and snake size on relative mass of prey (RPM). In all panels the areas below the curves represent the potential feeding performance space available to the snakes. In panels C and F, symbols show the sizes of whole prey consumed in the field, and the grey areas indicate the realized feeding performance spaces.

**Fig. 7** Three-dimensional feeding performance spaces delineated by RPM as a function of SVL and RPA for four crustacean-eating specialists consuming their normal prey (as in Fig. 4). Homalopsid data are from (Jayne et al. 2018). Overall, crayfish-eating snakes have larger performance spaces than crab-eating snakes, and juvenile snakes can usually consume prey with larger RPM for a given RPA.
