# Supplementary material for: Crayfish Eating in Snakes: Testing How Anatomy and Behavior Affect Prey Size and Feeding Performance
Source: Integr Org Biol. 2021 Jan 30;3(1):obab001. doi: 10.1093/iob/obab001 (PMC8023418; doi:10.1093/iob/obab001)
Supplement: obab001_Supplementary_Data [file obab001_supplementary_data.zip › IOB-2020-039.R2_Sup_Tables.docx]

**Table S1** Values of predicted maximal gape area with 95% confidence limits

| **Independent** | **Predicted values of maximal gape area (mm^2^)** | | |
| --- | --- | --- | --- |
| **Variable** | ***R. septemvittata*** |  | ***L. alleni*** |
| SVL = 165 mm | 58 (54, 62) |  | 82 (78, 85) |
| SVL = 328 mm | 177 (169, 185) |  | 243 (231, 256) |
| SVL = 490 mm | 341 (318, 366) |  | 461 (425, 501) |
| Mass = 2.6 g | 64 (59, 71) |  | 58 (54, 63) |
| Mass = 50 g | 326 (298, 356) |  | 274 (255, 294) |
| Mass = 98 g | 470 (419, 527) |  | 355 (354, 424) |

Predicted from sizes in common to both species in the sample used for the analysis of scaling relationships. Values in parentheses are lower and upper 95% confidence limits.

**Table S2** Mean values of prey handling times during laboratory trials

| **Behavior** | **Mean values ± SE (range)** | |
| --- | --- | --- |
|  | ***R. septemvittata* (*n* = 118)** | ***L. alleni* (*n* = 127)** |
| Attack time (s) | 57.7 ± 12.9 (0–711) | 14.4 ± 3.9 (0–326) |
| Holding (s) | 79.1 ± 7.1 (0–488) ^R, H^ | 2.4 ± 0.7 (0–49) |
| Lateral Jaw Walking (s) | 77.9 ± 8.3 (0–402) ^R^ | 50.4 ± 4.9 (1–293) ^R^ |
| Pre-swallow pause (s) | 20.6 ± 4.5 (0–283) ^R^ | 10.0 ± 3.0 (0–203) |
| Swallowing (s) | 137 ± 19.3 (2–1584) ^R^ | 214 ± 17.0 (12–1076) ^R^ |
| Swallow pause (s) | 23.0 ± 4.7 (0–350) ^R^ | 93.1 ± 10.8 (0–671) ^R, H^ |
| Handling time, HT (s) | 342 ± 31.7 (6–2376) ^R, H^ | 369 ± 28.9 (15–1823) ^R, H^ |

R and H indicate that when the duration of a behavior in the left column was a dependent variable in a univariate regression, it changed significantly with increased RPA and prey hardness, respectively (Table S4). Please note that values of different behaviors can occur in different trials and need not sum to the total handling time.

**Table S3** Final multiple regression models predicting total handling time and the occurrence of some behaviors

| **Model** | **Dependent Variable** | **Independent Variable** | **Coefficient** | ***P*** |
| --- | --- | --- | --- | --- |
|  | *R. septemvittata*  (*n* = 118) |  |  |  |
| 1 | log HT (s) | log RPA | 1.342 | <0.001 |
|  | (*R^2^* = 0.66) | Prey Hardness | 0.198 | <0.001 |
|  |  | Strike Location | 0.078 | 0.007 |
|  |  | Num. of Pinches | 0.094 | 0.025 |
|  |  | Constant | 0.102 | 0.517 |
|  |  |  |  |  |
| 2 | Presence of holding | log RPA | 0.770 | <0.001 |
|  | (*R^2^* = 0.42) | Prey Hardness | 0.312 | <0.001 |
|  |  | Strike Location | 0.084 | 0.007 |
|  |  | Constant | -0.665 | <0.001 |
|  | *L. alleni*  (*n* = 127) |  |  |  |
|  |  |  |  |  |
|  |  |  |  |  |
| 3 | log HT (s) | log RPA | 1.387 | <0.001 |
|  | (*R^2^* = 0.71) | Prey Hardness | 0.087 | <0.001 |
|  |  | Num. of Pinches | 0.039 | 0.007 |
|  |  | Prey Restraint | 0.057 | 0.001 |
|  |  | Constant | -0.049 | 0.769 |
|  |  |  |  |  |
| 4 | Type prey restraint | log RPA | 1.983 | <0.001 |
|  | (*R^2^* = 0.21) | Prey Hardness | 0.254 | <0.001 |
|  |  | Num. of Pinches | 0.174 | 0.017 |
|  |  | Constant | -2.057 | 0.016 |

RPA = crayfish area/ snake maximal gape area; prey hardness: 0 = soft, 1 = medium soft, 2 = hard; strike location: 1 = tail, 2 = junction of tail and abdomen, 3 = carapace; Num. of Pinches = number of pinches after crayfish capture; type of prey restraint: 0 = none, 1 = U-loop, 2 = pinning, 3 = coil.

**Table S4** Univariate regressions predicting handling times and behavior

| **Model** | **Independent Variable** | **Dependent Variable** | **Slope ±**  **95% CL** | **Intercept ± 95% CL** | ***R*^2^** | ***P*** |
| --- | --- | --- | --- | --- | --- | --- |
|  | *R. septemvittata (n* =118) | |  |  |  |  |
| 1 | log RPA | log HT | 1.329 ± 0.211 | 0.444 ± 0.306 | 0.57 | <0.001 |
| 1 | Prey Hardness | log HT | 0.172 ± 0.179 | 2.220 ± 0.147 | 0.02 | 0.059 |
| 1 | Num. Pinches | log HT | 0.168 ± 0.131 | 2.293 ± 0.089 | 0.04 | 0.012 |
| 1 | Location of Strike | log HT | 0.094 ± 0.095 | 2.139 ± 0.218 | 0.02 | 0.054 |
| 2 | log RPA | Presence of Holding | 0.729 ± 0.232 | -0.216 ± 0.336 | 0.24 | <0.001 |
| 2 | Prey Hardness | Presence of Holding | 0.281 ± 0.141 | 0.632 ± 0.116 | 0.11 | <0.001 |
| 2 | Strike Location | Presence of Holding | 0.094 ± 0.079 | 0.623 ± 0.180 | 0.04 | 0.019 |
|  | log RPA | Num. Unsuccessful Strikes | -0.788 ± 0.646 | 1.589 ± 0.936 | 0.04 | 0.017 |
|  | Prey Hardness | Num. Unsuccessful Strikes | -0.399 ± 0.365 | 0.737 ± 0.301 | 0.03 | 0.032 |
|  | Prey Hardness | Direction swallow | 0.338 ± 0.164 | 0.500 ± 0.135 | 0.12 | <0.001 |
|  | log RPA | Hold Time | 111.9 ± 49.94 | -80.33 ± 72.31 | 0.15 | <0.001 |
|  | Prey Hardness | Hold Time | 51.20 ± 28.83 | 44.34 ± 23.74 | 0.10 | <0.001 |
|  | log RPA | Pre-Swallow Pause | 19.81 ± 18.48 | -22.49 ± 26.76 | 0.03 | 0.036 |
|  | log RPA | Lateral Jaw Walk Time | 172.3 ± 86.51 | -142.7 ± 125.3 | 0.12 | <0.001 |
|  | log RPA | Swallow Pause | 80.05 ± 33.02 | -91.02 ± 47.82 | 0.16 | <0.001 |
|  | log RPA | Swallow Time | 541.9 ± 128.7 | -612.1 ± 186.3 | 0.37 | <0.001 |
|  | log RPA | Presence of Biting | 0.618 ± 0.263 | -0.092 ± 0.381 | 0.15 | <0.001 |
|  | Prey Hardness | Presence of Biting | 0.309 ± 0.150 | 0.580 ± 0.124 | 0.12 | <0.001 |
|  | log RPA | Tail flip+escape (PreCap) | -0.318 ± 0.306 | 0.724 ± 0.443 | 0.03 | 0.042 |
|  | log RPA | Tail flip (PostCap) | 0.521 ± 0.323 | -0.377 ± 0.468 | 0.07 | 0.002 |
|  | Prey Hardness | Tail flip (PostCap) | 0.188 ± 0.186 | 0.237 ± 0.153 | 0.03 | 0.048 |
|  | Prey Hardness | Pinch (PostCap) | 0.236 ± 0.147 | 0.026 ± 0.121 | 0.08 | 0.002 |
|  | *L. alleni* (hard, *n* = 61) | |  |  |  |  |
|  | log RPA | Prey Restraint | 2.428 + 1.307 | -1.448 + 1.894 | 0.19 | <0.001 |
|  | *L. alleni* (soft, *n* = 66) | |  |  |  |  |
|  | log RPA | Prey Restraint | 2.027 + 1.592 | -1.802 + 2.563 | 0.09 | 0.013 |
|  | *L. alleni* (all, *n* = 127) | |  |  |  |  |
| 3 | log RPA | log HT | 1.262 ± 0.237 | 0.480 ± 0.364 | 0.47 | <0.001 |
| 3 | Prey Hardness | log HT | 0.038 ± 0.043 | 2.312 ± 0.121 | 0.02 | 0.082 |
| 3 | Num. Punches | log HT | 0.102 ± 0.045 | 2.287 ± 0.083 | 0.13 | <0.001 |
| 3 | Type prey restraint | log HT | 0.158 ± 0.048 | 2.127 ± 0.105 | 0.24 | <0.001 |
| 4 | log RPA | Prey Restraint | 1.437 ± 0.998 | -0.460 ± 1.533 | 0.05 | 0.005 |
| 4 | Prey Hardness | Prey Restraint | 0.189 ± 0.132 | 1.293 ± 0.375 | 0.05 | 0.005 |
| 4 | Num. of Pinches | Prey Restraint | 0.271 ± 0.146 | 1.430 ± 0.269 | 0.09 | <0.001 |
|  | log RPA | Strike Location | 0.802 ± 0.710 | 0.719 ± 1.091 | 0.03 | 0.027 |
|  | log RPA | Lateral Jaw Walk Time | 117.8 ± 57.69 | -118.9 ± 88.61 | 0.11 | <0.001 |
|  | log RPA | Direction Swallow | -0.217 ± 0.182 | 1.322 ± 0.280 | 0.03 | 0.020 |
|  | log RPA | Swallow Pause (s) | 226.6 ± 88.06 | -251.2 ± 135.3 | 0.17 | <0.001 |
|  | Prey Hardness | Swallow Pause (s) | 16.33 ± 12.47 | 55.81 ± 12.47 | 0.04 | 0.011 |
|  | log RPA | Swallow Time | 736.7 ± 188.5 | -812.7 ± 289.6 | 0.32 | <0.001 |
|  | Prey Hardness | Presence of Biting | 0.057 ± 0.047 | 0.161 ± 0.133 | 0.04 | 0.017 |
|  | log RPA | Tail flip+escape (PreCap) | -0.292 ± 0.266 | 0.578 ± 0.408 | 0.03 | 0.031 |
|  | log RPA | Tail flip (PostCap) | 0.889 ± 0.364 | -0.887 ± 0.558 | 0.15 | <0.001 |
|  | Prey Hardness | Tail flip (PostCap) | -0.052 ± 0.052 | 0.590 ± 0.146 | 0.03 | 0.037 |
|  | log RPA | Tail flip+escape (PostCap) | 0.163 ± 0.152 | -0.209 ± 0.233 | 0.03 | 0.035 |
|  | Prey Hardness | Tail flip+escape (PostCap) | -0.026 ± 0.020 | 0.099 ± 0.057 | 0.04 | 0.010 |
|  | Prey Hardness | Pinch (PostCap) | 0.088 ± 0.050 | 0.279 ± 0.142 | 0.08 | 0.001 |

Independent variables that were also significant in multiple regressions are labelled with the number of the multiple regressions that are in Table S2. Variable abbreviations include: RPA, crayfish area/ snake maximal gape area; prey hardness (0 = soft, 1 = medium soft, 2 = hard); HT = handling time; presence of holding (0 = no holding, 1 = holding); prey restraint (0 = no body restraint, 1 = U-loop, 2 = body pin, 3 = coil); presence of biting (0 = no biting, 1 = > 1 bite); direction swallow (0 = head first, 1 = tail first); PreCap = crayfish behavior pre-capture; PostCap = crayfish behavior post-capture.

**Table S5** ANCOVA results for morphology and behaviors comparing the two species and sexes within a species

| Factor | Covariate | Dependent  Variable | Effect | Factor  *F_DF­_ (P)* | Covariate  X Factor Interaction  *F_DF­_ (P)* |
| --- | --- | --- | --- | --- | --- |
| Species | log Mass (g) | log SVL (mm) | La > Rs | 402.71_1,54_ (<0.001) | 0.62_1,53_ (0.436) |
| Species | log Gape Area (mm^2^) | log SVL (mm) | La > Rs | 123.15_1,54_ (<0.001) | 0.31_1,53_ (0.580) |
| Species | log Gape Area (mm^2^) | log Mass (g) | Rs > La | 15.02_1.54_ (<0.001) | 0.82_1,54_ (0.371) |
| Sex (Rs) | log Mass (g) | log SVL (mm) | M > F | 5.71_1,24_ (0.025) | 0.42_1,23_ (0.522) |
| Sex (Rs) | log Gape Area (mm^2^) | log SVL (mm) | F = M | 1.71_1,23_ (0.203) | 0.07_1,23_ (0.797) |
| Sex (Rs) | log Gape Area (mm^2^) | log Mass (g) | F > M | 5.45_1,24_ (0.028) | 1.23_1,23_ (0.729) |
| Sex (La) | log Mass (g) | log SVL (mm) | F = M | 0.780_1,27_ (0.385) | 1.60_1,26_ (0.218) |
| Sex (La) | log Gape Area (mm^2^) | log SVL (mm) | n/a | n/a | 4.70_1,26_ (0.040) |
| Sex (La) | log Gape Area (mm^2^) | log Mass (g) | F = M | 0.01_1,27_ (0.915) | 0.46_1,26_ (0.504) |
| Species | Log RPA (all) | Attack Time (s) | Rs > La | 13.08_1,242_ (<0.001) | 2.67_1,241_ (0.103) |
| Species | log RPA (all) | Num. Missed Strikes | Rs > La | 6.30_1,242_ (0.013) | 2.42_1,241_ (0.121) |
| Species | log RPA (all) | Strike location | Rs = La | 3.52_1,242_ (0.062) | 2.41_1,241_ (0.122) |
| Species | log RPA (all) | Num. of Bites | Rs > La | 5.10_1,242_ (0.025) | 0.695_1,241_ (0.405) |
| Species | log RPA (all) | Hold Time (s) | n/a | n/a | 18.2_1,241_ (<0.001) |
| Species | log RPA (all) | Lat Jaw Walk (s) | Rs > La | 18.56_1,242_ (<0.001) | 1.03_1,241_ (0.311) |
| Species | log RPA (all) | Orientation Swallow | n/a | n/a | 4.95_1,241_ (0.027) |
| Species | log RPA (all) | Direction Swallow | La > Rs | 33.60_1,242_ (<0.001) | 2.78_1,241_ (0.097) |
| Species | log RPA (all) | Pre-Swallow Pause | Rs > La | 8.11_1,242_ (0.005) | 3.51_1,241_ (0.062) |
| Species | log RPA (all) | Swallow Pause | n/a | n/a | 10.08_1,241_ (0.002) |
| Species | log RPA (all) | Swallow Time(s) | La > Rs | 9.64_1,242­_ (0.002) | 2.92_1,241_ (0.089) |
| Species | log RPA (all) | log HT (s) | Rs = La | 2.59_1,242_ (0.109) | 0.174_1,241_ (0.677) |
| Species | log RPA (molt) | log HT (s) | Rs > La | 27.9_1,181_ (<0.001) | 2.88_1,180_ (0.091) |
| Species | log RPA (hard/molt) | log HT (s) | La > Rs | 5.814_1,176_ (0.017) | 0.77_1,1785_ (0.381) |
| Species | log RPM (all) | log HT (s) | n/a | n/a | 7.97_1,241_ (0.005) |

Factors include: species (La = *L. alleni,* Rs *=* *R. septemvittata*); and sex (F = female, M = male). Covariates include: Gape Area = maximum gape area and diameter of both species; RPA = crayfish area/ snake maximal gape area; RPM = crayfish mass/snake mass. The parenthetical notation next to RPA and RPM indicate the prey hardness of (La/Rs): all = soft, medium, and hard-shell prey, soft = soft and medium prey, hard = hard-shell prey only. Dependent variables include: strike location (1 = tail, 2 = junction of tail and abdomen, 3 = carapace); Orientation swallow (1 = lateral, 2 = dorsal, 3 = ventral); direction swallow (0 = head first, 1 = tail first; HT = handling time). The effect column indicates which sex or species had larger values of the dependent variable n/a = equality of slopes failed and could not test for the effect of species or sex.
