# Supplementary material for: Crayfish Eating in Snakes: Testing How Anatomy and Behavior Affect Prey Size and Feeding Performance
Source: Integr Org Biol. 2021 Jan 30;3(1):obab001. doi: 10.1093/iob/obab001 (PMC8023418; doi:10.1093/iob/obab001)
Supplement: obab001_Supplementary_Data [file obab001_supplementary_data.zip › IOB-2020-039.R2_Sup_Tables.pdf]

Table S1 Values of predicted maximal gape area with 95% confidence limits

Independent Variable	Predicted values of maximal gape area (mm^2)	
	R. septemvittata	***L. alleni***
SVL = 165 mm	58 (54, 62)	82 (78, 85)
SVL = 328 mm	177 (169, 185)	243 (231, 256)
SVL = 490 mm	341 (318, 366)	461 (425, 501)
Mass = 2.6 g	64 (59, 71)	58 (54, 63)
Mass = 50 g	326 (298, 356)	274 (255, 294)
Mass = 98 g	470 (419, 527)	355 (354, 424)

Predicted from sizes in common to both species in the sample used for the analysis of scaling relationships. Values in parentheses are lower and upper 95% confidence limits.

Table S2 Mean values of prey handling times during laboratory trials

**Behavior**	**Mean values ± SE (range)**	
	***R. septemvittata* (*n* = 118)**	***L. alleni* (*n* = 127)**
Attack time (s)	57.7 ± 12.9 (0–711)	14.4 ± 3.9 (0–326)
Holding (s)	79.1 ± 7.1 (0–488) [R, H]	2.4 ± 0.7 (0–49)
Lateral Jaw Walking (s)	77.9 ± 8.3 (0–402) [R]	50.4 ± 4.9 (1–293) [R]
Pre-swallow pause (s)	20.6 ± 4.5 (0–283) [R]	10.0 ± 3.0 (0–203)
Swallowing (s)	137 ± 19.3 (2–1584) [R]	214 ± 17.0 (12–1076) [R]
Swallow pause (s)	23.0 ± 4.7 (0–350) [R]	93.1 ± 10.8 (0–671) [R, H]
Handling time, HT (s)	342 ± 31.7 (6–2376) [R, H]	369 ± 28.9 (15–1823) [R, H]

R and H indicate that when the duration of a behavior in the left column was a dependent variable in a univariate regression, it changed significantly with increased RPA and prey hardness, respectively (Table S4). Please note that values of different behaviors can occur in different trials and need not sum to the total handling time.

Table S3 Final multiple regression models predicting total handling time and the occurrence of some behaviors

Model	Dependent Variable	Independent Variable	Coefficient	*P*
	R. septemvittata (*n* = 118)			
1	log HT (s)	log RPA	1.342	<0.001
	(R^2 = 0.66)	Prey Hardness	0.198	<0.001
		Strike Location	0.078	0.007
		Num. of Pinches	0.094	0.025
		Constant	0.102	0.517
2	Presence of holding	log RPA	0.770	<0.001
	(R^2 = 0.42)	Prey Hardness	0.312	<0.001
		Strike Location	0.084	0.007
		Constant	-0.665	<0.001
	L. alleni (*n* = 127)			
3	log HT (s)	log RPA	1.387	<0.001
	(R^2 = 0.71)	Prey Hardness	0.087	<0.001
		Num. of Pinches	0.039	0.007
		Prey Restraint	0.057	0.001
		Constant	-0.049	0.769
4	Type prey restraint	log RPA	1.983	<0.001
	(R^2 = 0.21)	Prey Hardness	0.254	<0.001
		Num. of Pinches	0.174	0.017
		Constant	-2.057	0.016

RPA = crayfish area/ snake maximal gape area; prey hardness: 0 = soft, 1 = medium soft, 2 = hard; strike location: 1 = tail, 2 = junction of tail and abdomen, 3 = carapace; Num. of Pinches = number of pinches after crayfish capture; type of prey restraint: 0 = none, 1 = U-loop, 2 = pinning, 3 = coil.

Table S4 Univariate regressions predicting handling times and behavior

Model	Independent Variable	Dependent Variable	Slope ± 95% CL	Intercept ± 95% CL	R^2	*P*
	R. septemvittata (n =118)					
1	log RPA	log HT	1.329 ± 0.211	0.444 ± 0.306	0.57	<0.001
1	Prey Hardness	log HT	0.172 ± 0.179	2.220 ± 0.147	0.02	0.059
1	Num. Pinches	log HT	0.168 ± 0.131	2.293 ± 0.089	0.04	0.012
1	Location of Strike	log HT	0.094 ± 0.095	2.139 ± 0.218	0.02	0.054
2	log RPA	Presence of Holding	0.729 ± 0.232	-0.216 ± 0.336	0.24	<0.001
2	Prey Hardness	Presence of Holding	0.281 ± 0.141	0.632 ± 0.116	0.11	<0.001
2	Strike Location	Presence of Holding	0.094 ± 0.079	0.623 ± 0.180	0.04	0.019
	log RPA	Num. Unsuccessful Strikes	-0.788 ± 0.646	1.589 ± 0.936	0.04	0.017
	Prey Hardness	Num. Unsuccessful Strikes	-0.399 ± 0.365	0.737 ± 0.301	0.03	0.032
	Prey Hardness	Direction swallow	0.338 ± 0.164	0.500 ± 0.135	0.12	<0.001
	log RPA	Hold Time	111.9 ± 49.94	-80.33 ± 72.31	0.15	<0.001
	Prey Hardness	Hold Time	51.20 ± 28.83	44.34 ± 23.74	0.10	<0.001
	log RPA	Pre-Swallow Pause	19.81 ± 18.48	-22.49 ± 26.76	0.03	0.036
	log RPA	Lateral Jaw Walk Time	172.3 ± 86.51	-142.7 ± 125.3	0.12	<0.001
	log RPA	Swallow Pause	80.05 ± 33.02	-91.02 ± 47.82	0.16	<0.001
	log RPA	Swallow Time	541.9 ± 128.7	-612.1 ± 186.3	0.37	<0.001
	log RPA	Presence of Biting	0.618 ± 0.263	-0.092 ± 0.381	0.15	<0.001
	Prey Hardness	Presence of Biting	0.309 ± 0.150	0.580 ± 0.124	0.12	<0.001
	log RPA	Tail flip+escape (PreCap)	-0.318 ± 0.306	0.724 ± 0.443	0.03	0.042
	log RPA	Tail flip (PostCap)	0.521 ± 0.323	-0.377 ± 0.468	0.07	0.002
	Prey Hardness	Tail flip (PostCap)	0.188 ± 0.186	0.237 ± 0.153	0.03	0.048
	Prey Hardness	Pinch (PostCap)	0.236 ± 0.147	0.026 ± 0.121	0.08	0.002
	L. alleni (hard, *n* = 61)					
	log RPA	Prey Restraint	2.428 ± 1.307	-1.448 ± 1.894	0.19	<0.001
	L. alleni (soft, *n* = 66)					
	log RPA	Prey Restraint	2.027 ± 1.592	-1.802 ± 2.563	0.09	0.013
	L. alleni (all, *n* = 127)					
3	log RPA	log HT	1.262 ± 0.237	0.480 ± 0.364	0.47	<0.001
3	Prey Hardness	log HT	0.038 ± 0.043	2.312 ± 0.121	0.02	0.082
3	Num. Punches	log HT	0.102 ± 0.045	2.287 ± 0.083	0.13	<0.001
3	Type prey restraint	log HT	0.158 ± 0.048	2.127 ± 0.105	0.24	<0.001
4	log RPA	Prey Restraint	1.437 ± 0.998	-0.460 ± 1.533	0.05	0.005
4	Prey Hardness	Prey Restraint	0.189 ± 0.132	1.293 ± 0.375	0.05	0.005
4	Num. of Pinches	Prey Restraint	0.271 ± 0.146	1.430 ± 0.269	0.09	<0.001
	log RPA	Strike Location	0.802 ± 0.710	0.719 ± 1.091	0.03	0.027
	log RPA	Lateral Jaw Walk Time	117.8 ± 57.69	-118.9 ± 88.61	0.11	<0.001
	log RPA	Direction Swallow	-0.217 ± 0.182	1.322 ± 0.280	0.03	0.020
	log RPA	Swallow Pause (s)	226.6 ± 88.06	-251.2 ± 135.3	0.17	<0.001
	Prey Hardness	Swallow Pause (s)	16.33 ± 12.47	55.81 ± 12.47	0.04	0.011
	log RPA	Swallow Time	736.7 ± 188.5	-812.7 ± 289.6	0.32	<0.001

Prey Hardness	Presence of Biting	0.057 ± 0.047	0.161 ± 0.133	0.04	0.017
log RPA	Tail flip+escape (PreCap)	-0.292 ± 0.266	0.578 ± 0.408	0.03	0.031
log RPA	Tail flip (PostCap)	0.889 ± 0.364	-0.887 ± 0.558	0.15	<0.001
Prey Hardness	Tail flip (PostCap)	-0.052 ± 0.052	0.590 ± 0.146	0.03	0.037
log RPA	Tail flip+escape (PostCap)	0.163 ± 0.152	-0.209 ± 0.233	0.03	0.035
Prey Hardness	Tail flip+escape (PostCap)	-0.026 ± 0.020	0.099 ± 0.057	0.04	0.010
Prey Hardness	Pinch (PostCap)	0.088 ± 0.050	0.279 ± 0.142	0.08	0.001

Independent variables that were also significant in multiple regressions are labelled with the number of the multiple regressions that are in Table S2. Variable abbreviations include: RPA, crayfish area/ snake maximal gape area; prey hardness (0 = soft, 1 = medium soft, 2 = hard); HT = handling time; presence of holding (0 = no holding, 1 = holding); prey restraint (0 = no body restraint, 1 = U-loop, 2 = body pin, 3 = coil); presence of biting (0 = no biting, 1 = ≥ 1 bite); direction swallow (0 = head first, 1 = tail first); PreCap = crayfish behavior pre-capture; PostCap = crayfish behavior post-capture.

Table S5 ANCOVA results for morphology and behaviors comparing the two species and sexes within a species

Factor	Covariate	Dependent Variable	Effect	Factor F_{DF} (*P*)	Covariate X Factor Interaction F_{DF} (*P*)
Species	log Mass (g)	log SVL (mm)	La > Rs	$402.71_{1,54}$ (<0.001)	$0.62_{1,53}$ (0.436)
Species	log Gape Area (mm^2)	log SVL (mm)	La > Rs	$123.15_{1,54}$ (<0.001)	$0.31_{1,53}$ (0.580)
Species	log Gape Area (mm^2)	log Mass (g)	Rs > La	$15.02_{1.54}$ (<0.001)	$0.82_{1,54}$ (0.371)
Sex (Rs)	log Mass (g)	log SVL (mm)	M > F	$5.71_{1,24}$ (0.025)	$0.42_{1,23}$ (0.522)
Sex (Rs)	log Gape Area (mm^2)	log SVL (mm)	F = M	$1.71_{1,23}$ (0.203)	$0.07_{1,23}$ (0.797)
Sex (Rs)	log Gape Area (mm^2)	log Mass (g)	F > M	$5.45_{1,24}$ (0.028)	$1.23_{1,23}$ (0.729)
Sex (La)	log Mass (g)	log SVL (mm)	F = M	$0.780_{1,27}$ (0.385)	$1.60_{1,26}$ (0.218)
Sex (La)	log Gape Area (mm^2)	log SVL (mm)	n/a	n/a	$4.70_{1,26}$ (0.040)
Sex (La)	log Gape Area (mm^2)	log Mass (g)	F = M	$0.01_{1,27}$ (0.915)	$0.46_{1,26}$ (0.504)
Species	Log RPA (all)	Attack Time (s)	Rs > La	$13.08_{1,242}$ (<0.001)	$2.67_{1,241}$ (0.103)
Species	log RPA (all)	Num. Missed Strikes	Rs > La	$6.30_{1,242}$ (0.013)	$2.42_{1,241}$ (0.121)
Species	log RPA (all)	Strike location	Rs = La	$3.52_{1,242}$ (0.062)	$2.41_{1,241}$ (0.122)
Species	log RPA (all)	Num. of Bites	Rs > La	$5.10_{1,242}$ (0.025)	$0.695_{1,241}$ (0.405)
Species	log RPA (all)	Hold Time (s)	n/a	n/a	$18.2_{1,241}$ (<0.001)
Species	log RPA (all)	Lat Jaw Walk (s)	Rs > La	$18.56_{1,242}$ (<0.001)	$1.03_{1,241}$ (0.311)
Species	log RPA (all)	Orientation Swallow	n/a	n/a	$4.95_{1,241}$ (0.027)
Species	log RPA (all)	Direction Swallow	La > Rs	$33.60_{1,242}$ (<0.001)	$2.78_{1,241}$ (0.097)
Species	log RPA (all)	Pre-Swallow Pause	Rs > La	$8.11_{1,242}$ (0.005)	$3.51_{1,241}$ (0.062)
Species	log RPA (all)	Swallow Pause	n/a	n/a	$10.08_{1,241}$ (0.002)
Species	log RPA (all)	Swallow Time(s)	La > Rs	$9.64_{1,242}$ (0.002)	$2.92_{1,241}$ (0.089)
Species	log RPA (all)	log HT (s)	Rs = La	$2.59_{1,242}$ (0.109)	$0.174_{1,241}$ (0.677)
Species	log RPA (molt)	log HT (s)	Rs > La	$27.9_{1,181}$ (<0.001)	$2.88_{1,180}$ (0.091)
Species	log RPA (hard/molt)	log HT (s)	La > Rs	$5.814_{1,176}$ (0.017)	$0.77_{1,1785}$ (0.381)
Species	log RPM (all)	log HT (s)	n/a	n/a	$7.97_{1,241}$ (0.005)

Factors include: species (La = *L. alleni,* Rs = *R. septemvittata*); and sex (F = female, M = male). Covariates include: Gape Area = maximum gape area and diameter of both species; RPA = crayfish area/ snake maximal gape area; RPM = crayfish mass/snake mass. The parenthetical notation next to RPA and RPM indicate the prey hardness of (La/Rs): all = soft, medium, and hard-shell prey, soft = soft and medium prey, hard = hard-shell prey only. Dependent variables include: strike location (1 = tail, 2 = junction of tail and abdomen, 3 = carapace); Orientation swallow (1 = lateral, 2 = dorsal, 3 = ventral); direction swallow (0 = head first, 1 = tail first; HT = handling time). The effect column indicates which sex or species had larger values of the dependent variable n/a = equality of slopes failed and could not test for the effect of species or sex.
